# Anaplastic thyroid cancer: How far can we go?

**DOI:** 10.17179/excli2020-1302

**Published:** 2020-06-15

**Authors:** Mariana Amaral, Ricardo A. Afonso, M. Manuela Gaspar, Catarina Pinto Reis

**Affiliations:** 1 Research Institute for Medicines (iMed.ULisboa), Faculdade de Farmácia, Universidade de Lisboa, Lisboa, Portugal; 2 CEDOC, NOVA Medical School, Faculdade de Ciências Médicas (NMS/FCM), Universidade Nova de Lisboa, Lisboa, Portugal; 3 Área de Ensino e Investigação em Ciências Funcionais e Alvos Terapêuticos, NOVA Medical School, Faculdade de Ciências Médicas (NMS|FCM), Universidade Nova de Lisboa, Lisboa, Portugal; 4 Departamento de Física, Faculdade de Ciências e Tecnologia, Universidade Nova de Lisboa, Lisboa, Portugal; 5 IBEB, Institute of Biophysics and Biomedical Engineering, Faculdade de Ciências, Universidade de Lisboa, Portugal

**Keywords:** anaplastic thyroid cancer, treatments and obstacles, challenges for innovative therapies

## Abstract

Globally, thyroid cancer accounts for 2 % of all cancer diagnoses, and can be classified as well-differentiated or undifferentiated. Currently, differentiated thyroid carcinomas have good prognoses, and can be treated with a combination of therapies, including surgical thyroidectomy, radioactive iodine therapy and hormone-based therapy. On the other hand, anaplastic thyroid carcinoma, a subtype of undifferentiated thyroid carcinoma characterized by the loss of thyroid-like phenotype and function, does not respond to either radioactive iodine or hormone therapies. In most cases, anaplastic thyroid carcinomas are diagnosed in later stages of the disease, deeming them inoperable, and showing poor response rates to systemic chemotherapy. Recently, treatment courses using multiple-target agents are being explored and clinical trials have shown very promising results, such as overall survival rates, progression-free survival and tumor shrinkage. This review is focused on thyroid carcinomas, with particular focus on anaplastic thyroid carcinoma, exploring its undifferentiated nature. Special interest will be given to the treatment approaches currently available and respective obstacles or drawbacks. Our purpose is to contribute to understand why this malignancy presents low responsiveness to current treatments, while overviewing novel therapies and clinical trials.

## Introduction

The thyroid is a butterfly-shaped gland composed by two lobes, located medially in front of the neck, below the larynx and posteriorly to the tracheal thyroid cartilage (Stathatos, 2006[77]). This gland has a functional unit, the thyroid follicle, a cystic structure composed of a single layer of follicular cells (Stathatos, 2012[78]). These units store thyroglobulin, involved in the synthesis of thyroid hormones (*i.e.,* tri-iodothyronine, T_3_; and tetraiodothyronine, T_4_) (Rousset et al., 2000[59]; Stathatos, 2012[78]). Thyroid hormone production and secretion are controlled by the hypothalamus-pituitary axis, comprising the release of thyrotropin-releasing hormone (TRH) from the hypothalamus, and thyroid-stimulating hormone (TSH), from the pituitary (Opitz et al., 2009[50]; Stathatos, 2012[78]). Upon release by the pituitary, TSH binds to the TSH receptor (TSHR) of the follicular cells' membrane, stimulating the synthesis and release of the thyroid hormones (Opitz et al., 2009[50]; Stathatos, 2012[78]). Regulation of thyroid function is achieved almost entirely by a negative feedback mechanism carried out by T_3_ and T_4_ on hypothalamus and pituitary. 

T_3_ and T_4_ are known to play important roles in the human body, such as promoting the growth and differentiation of many tissues, as well as energy and metabolic homeostasis, due to their involvement in different metabolic pathways (Jugan et al., 2010[24]). Moreover, the thyroid gland also comprises neural-crest derived parafollicular C-cells, located in-between thyroid follicles and responsible for calcitonin secretion (Khan and Farhana, 2020[27]). This hormone promotes calcium and phosphate deposition in different tissues (Khan and Farhana, 2020[27]). 

Although thyroid cancers account for only 2.1 % of all cancers diagnosed worldwide, it is one of the most frequent endocrine malignancies (Gimm, 2001[15]; Kitahara and Sosa, 2016[28]; Nikiforova and Nikiforov, 2008[48]). As with other malignancies, thyroid cancers differ in their morphology, invasiveness and molecular profile (Lin, 2011[39]; Nikiforova and Nikiforov, 2008[48]). 

Taking into account their histopathology, thyroid carcinomas can be classified as well-differentiated (medullary, papillary and follicular thyroid carcinoma) or undifferentiated (anaplastic thyroid carcinoma) (Kondo et al., 2006[30]; Lin, 2011[39]; Soares et al., 2011[75]), as summarized in Table 1(Tab. 1).

## Epidemiology and Prognosis

Papillary thyroid carcinoma accounts for the largest portion of thyroid carcinomas (70-80 %), being the least aggressive due to slowly-forming metastasis and low invasiveness (Nguyen et al., 2015[46]). Follicular thyroid carcinoma is a more aggressive subtype of well-differentiated thyroid carcinoma, due to its usual later diagnosis, and accounts for 10 % of thyroid malignancies (D'Avanzo et al., 2004[11]). Medullary thyroid carcinoma accounts for 5-10 % of thyroid malignancies, originates from parafollicular C-cells, is associated with a mutation of the *RET* proto-oncogene and can be sporadic or familial (25 % of medullary thyroid carcinomas) (Leboulleux et al., 2004[33]). Another form of hereditary thyroid carcinoma is familial non-medullary thyroid carcinoma, englobing all hereditary thyroid carcinomas originating from thyroid follicular cells (Nosé, 2008[49]; Robenshtok et al., 2011[56]). Although familial non-medullary thyroid carcinoma is inherited through an autosomal dominant pattern, the associated mutated genes are not yet identified (Robenshtok et al., 2011[56]). 

Anaplastic thyroid carcinoma is a form of undifferentiated thyroid carcinoma, which although rare (<2 %), is one of the most lethal malignancies, being characterized by high aggressiveness, due to both fast growth and strong invasiveness, as well as low responsiveness to most therapies currently available (Kebebew et al., 2005[25]; Wiseman et al., 2003[85]). Moreover, although anaplastic thyroid carcinoma arises from thyroid follicular cells, these cells loose its thyroid-like features, leading to very poor prognosis (Lang and Lo, 2007[32]). The overall 5-year survival rate upon anaplastic thyroid carcinoma diagnosis is lower than 10 %, and most patients do not live longer than a few months after diagnosis (Liu et al., 2016[40]).

These two groups of malignancies have different aggressiveness. Indeed, well-differentiated thyroid carcinomas are known to be more manageable, with higher survival rates, whereas undifferentiated thyroid carcinomas are known to be more aggressive, with higher invasiveness and poorer prognosis, normally non-operable and having poor treatment response rates (DeLellis, 2006[12]). Well-differentiated thyroid carcinomas include malignancies derived from the thyroids' follicular cells, such as papillary and follicular thyroid carcinomas (Soares et al., 2004[76]). Anaplastic thyroid carcinoma is an undifferentiated subtype with very poor survival prognosis, estimated to be 3 to 5 months after diagnosis, and survival rates of 10-20 % and less than 5 % after 1 and 10 years, respectively (Nagaiah et al., 2011[44]). Although this rare tumor has an incidence of only 1-2 persons per million per year, it is responsible for 40 % of all thyroid cancer deaths (Green et al., 2006[18]; Tiedje et al., 2018[81]). The very poor prognosis is associated to anaplastic thyroid carcinoma only being detectable by the current diagnostic tools at advanced stages and, furthermore, being unresponsive to the current treatments available (Lin, 2011[39]). 

In similarity to what is seen for malignancies of other tissues and/or organs, there are risk factors associated with the increased chances of developing thyroid carcinomas. Such risk factors include radiation exposure to the chest or neck area, abnormal iodine intake leading to iodine deficits, previously-existing thyroid pathologies (*i.e.,* goiter and Hashimoto's Thyroiditis) and metabolic disorders (*i.e.,* diabetes and obesity) (Liu et al., 2017[41]). There are some etiologic factors specifically associated with the development of anaplastic thyroid carcinoma, both as primary disease or by dedifferentiation of other thyroid malignancies. Such etiological factors include irradiation and abnormal TSH levels (Khairy, 2009[26]). The biggest risk factor for developing this rare undifferentiated carcinoma seems to be prior history of goiter, both of self and familial (Nagaiah et al., 2011[44]). Furthermore, risk factors generally include previous history of other thyroid malignancies, as these can give rise to anaplastic thyroid carcinoma through dedifferentiation (Dackiw, 2010[10]; Khairy, 2009[26]). Anaplastic thyroid carcinoma seems to occur most frequently in the elderly, being diagnosed at around 65-72 years old (Zivaljevic et al., 2014[87]). Generally, both well-differentiated and anaplastic thyroid carcinomas affect women 2 to 3 times more than men (Tuttle et al., 2010[82]). 

## Pathophysiology and Histology

As previously mentioned, based on their histology and behavior, thyroid carcinomas can be subcategorized in well-differentiated and undifferentiated (anaplastic) thyroid carcinomas. Regarding histology, well-differentiated thyroid carcinoma arises from the thyroids' follicular cells and can be classified as papillary, if a papillary pattern is seen, or follicular, if a follicular pattern is found (Shah, 2015[65]). Although both patterns may be present, classification is based on the most prevalent pattern observed (Shah, 2015[65]). Furthermore, well-differentiated thyroid carcinoma aggressiveness is determined by assessing the presence of capsular and/or blood vessels invasion (Filetti et al., 2019[13]). Usually, papillary thyroid carcinomas present as an encapsulated mass, not invasive, whereas the follicular subtype presents high invasiveness, of both capsule and blood vessels. Papillary thyroid carcinomas can be further distinguished into two classes according to molecular profiling: *BRAF*-predominant; and *RAS*-predominant, the last associated with increased aggressiveness (Filetti et al., 2019[13]). 

Usually, thyroid carcinoma staging is determined by age, histology, size, extra-glandular invasion and presence of distance metastasis (Cady, 1998[9]). Regardless of the patient's and tumor status based on the mentioned characteristics, anaplastic thyroid carcinomas are always classified as stage IV (Kebebew et al., 2005[25]; Nguyen et al., 2015[46]; Tahara et al., 2017[80]). Then, by assessing different parameters, it can be sub-classified as: stage IVA, if it is confined to the thyroid; stage IVB, when there is extra thyroidal disease; or stage IVC, once distant metastasis are present (Ranganath et al., 2015[55]).

Anaplastic thyroid carcinoma is often clinically characterized as a large palpable rapidly growing mass, causing symptoms such as hoarseness, dysphagia, dyspnea, and in advanced cases, superior vena cava syndrome and Horner's syndrome (Cabanillas et al., 2016[8]; Wein and Weber, 2011[84]). Histologically, the characteristic cells of this tumor are known to have undergone epithelial-mesenchymal cell phenotype transition (Lin, 2011[39]). Furthermore, histological findings may follow one of three patterns according to the main cellular population present being giant, spindle or squamous cells (Cabanillas et al., 2016[8]; Wein and Weber, 2011[84]). This leads to uncertainty of the organ of origin, culminating in delays in diagnostic and in initiation of treatment (Cabanillas et al., 2016[8]; Wein and Weber, 2011[84]). Although these histological differences may be present, they do not significantly influence prognosis (Are and Shaha, 2006[4]). Macroscopically, regardless of its cellular heterogeneity, anaplastic thyroid carcinoma presents characteristically as large light tan color tumors, with marked invasiveness and mitotic activity, high proliferation, presence of hemorrhage and large areas of necrotic tissue, but decreased apoptosis (Are and Shaha, 2006[4]). 

Although this undifferentiated malignancy can arise primarily, there is clinical, pathologic and epidemiologic evidences supporting that it can originate from the dedifferentiation of previously-existing well-differentiated thyroid carcinomas (Neff et al., 2008[45]; Nikiforov, 2004[47]). Such evidence includes the fact that these tumors can coexist and that some treated well-differentiated thyroid carcinomas relapse as anaplastic thyroid carcinomas (Santarpia et al., 2008[62]). Furthermore, the genetic modifications and oncogenes that give rise to follicular and/or papillary thyroid carcinomas are also observed in anaplastic thyroid carcinomas (Neff et al., 2008[45]; Nikiforov, 2004[47]; Wang et al., 2007[83]). Moreover, anaplastic thyroid carcinoma harbors some characteristic genetic features (Ragazzi et al., 2014[54]). For example, gain of function mutations of the *PIK3CA* gene are frequently seen in anaplastic thyroid carcinoma, but not in well-differentiated thyroid carcinomas (Ragazzi et al., 2014[54]). Mutations in the gene encoding β-Catenin, *CTNNB1*, are commonly associated with epithelial-mesenchymal transition, which has been speculated as being one of the main processes behind this malignancy pathogenesis (DeLellis, 2006[12]; Ragazzi et al., 2014[54]). The previously mentioned mutations are gain of function of important oncogenes, but the loss of function and inactivation of tumor suppressor genes are also present in anaplastic thyroid carcinoma (Ragazzi et al., 2014[54]; Salvatore et al., 2007[61]). Such genes include *p53* and *PTEN*, both negative regulators of proliferation and inducers of apoptosis (DeLellis, 2006[12]; Quiros et al., 2005[53]; Salvatore et al., 2007[61]). Thus, the inactivity of these genes lead to increased aggressiveness, and are present in this non-differentiated tumor (Ragazzi et al., 2014[54]). Anaplastic thyroid carcinoma shares mutations with follicular and/ or papillary thyroid carcinomas, such as point mutations in *BRAF* and *RAS*, but these mutations are more common in the differentiated subtypes (Antonelli et al., 2008[3]; Ragazzi et al., 2014[54]; Salvatore et al., 2007[61]). Furthermore, the overexpression of receptors, such as epidermal growth factor receptor (EGFR), are not only characteristic of anaplastic thyroid carcinoma but also of thyroid and primary thyroid carcinomas dedifferentiation or anaplastic carcinoma transformation (Fisher et al., 2013[14]; Landriscina et al., 2011[31]).

## Diagnosis

Well-differentiated thyroid carcinomas, such as follicular or papillary thyroid carcinomas, are usually asymptomatic and are diagnosed upon physical and/or ultrasonography examination (Paschke et al., 2015[52]). In rare occasions, well-differentiated thyroid carcinomas may present symptoms such as a palpable and growing neck mass, hoarseness, dysphagia and/or with cervical lymph-node metastases (Paschke et al., 2015[52]). 

Anaplastic thyroid carcinoma generally presents more serious symptoms, including hoarseness, airway distress, dyspnea and dysphagia, caused by a fast growing neck mass (Akaishi et al., 2011[1]; Shaha, 2008[66]).

In order to classify a thyroid nodule as malignant or benign, TSH serum levels are evaluated, and a combination of histologic, cytologic and imaging techniques are used. TSH serum levels allow to differentiate between hyperfunctioning and non-functioning nodules (Nguyen et al., 2015[46]). Thyroid carcinomas often present non-functioning thyroid nodules, and thus, other tests are generally required (Nguyen et al., 2015[46]). 

Definitive diagnostic is usually achieved by fine-needle aspiration biopsy and/or high-resolution ultrasonography (Huang et al., 2015[22]; Lewinski et al., 2000[35]; Nguyen et al., 2015[46]). Papillary, medullary and anaplastic thyroid carcinomas are diagnosed according to the results of these examinations, but additional histological tests can be necessary to differentiate between follicular thyroid carcinoma and benign follicular thyroid adenomas (Sherman, 2003[68]).

## Current Treatment Approaches

Currently, thyroid cancer is treated by using a combination of radioactive iodine therapy, thyroid hormone suppression therapy and surgery. Nevertheless, the chosen treatment is defined according to different factors, such as the subtype of cancer and stage of disease (Nguyen et al., 2015[46]). 

Total or partial surgical resection of the thyroid gland remains one of the first options for both well-differentiated and undifferentiated thyroid carcinomas, although in the latter total thyroidectomy is unusual due to invasiveness of the disease (Cabanillas et al., 2016[8]; Giuffrida and Gharib, 2000[16]).

Therapy with radioactive iodine has been used to treat thyroid cancer since 1946 (Lee, 2010[34]). For radioactive iodine therapy to be effective, a high level of thyroid-stimulating hormone (TSH or thyrotropin) must be present in the blood to promote its uptake/or absorption. To synthetize thyroid hormones, the thyroid follicular cells need to uptake iodine, a substrate required for the synthesis of these hormones. Iodine is internalized by the follicular cells through iodine symporter channels. Inside the follicular cells, the iodine is oxidized and bound to tyrosyl residues of thyroglobulin, giving rise to tri-iodothyronine (T_3_) and tetraiodothyronine (T_4_), with three and four atoms of iodine, respectively (Biondi et al., 2005[5]; Ross, 2011[58]). When in the presence of Radioactive Iodine (^131^I) instead of normal iodine, the ^131^I undergoes the previously described processes in the follicular cells, leading to tissue necrosis mediated by its beta emissions. As a result, this necrosis will lead to the ablation of the functional tissues of the thyroid gland (Ross, 2011[58]; Schlumberger et al., 2014[64]). Conventionally, this therapy is used after surgery, either as an adjuvant therapy or to treat any tumoral residual tissue (Jonklaas et al., 2006[23]; Sawka et al., 2004[63]). Undifferentiated and medullary thyroid carcinomas do not respond to this therapy as they are characterized by a lack of expression of thyroid cell markers and behavior, being unable to uptake iodine and consequently produce T_3_ and T_4_ (Cabanillas et al., 2016[8]; Sherman, 2003[68]).

Usually, life-long thyroid hormone therapy (THST) is used to treat well-differentiated and medullary thyroid carcinomas after thyroidectomy and radioactive iodine therapy to prevent thyroid-stimulating hormone (TSH)-dependent proliferation of any residual well-differentiated thyroid cancer cells (Brabant, 2008[6]; McGriff et al., 2002[42]; Sherman, 2003[68]). Physiologically, TSH release by the pituitary is inhibited by high serum levels of T_3 _and T_4_, *i.e.*, through a negative feedback mechanism (Biondi et al., 2005[5]). Thus, this therapy involves the administration of T_3_ and T_4_, increasing serum levels of these two hormones and inhibiting pituitary TSH release (Biondi et al., 2005[5]). Moreover, besides inhibiting TSH-dependent proliferation of cancer cells, THST also corrects the surgically-induced hypothyroidism of patients who undergo total or partial thyroid resection (Biondi et al., 2005[5]). 

Medullary thyroid carcinomas and undifferentiated thyroid carcinomas are usually unresponsive to the conventional course of therapy used for well-differentiated thyroid carcinomas: radioactive iodine therapy, THST and surgery (Sherman, 2010[68]). For this reason, systemic chemotherapy is used for the treatment of the mentioned malignancies, and also for the treatment of non-resectable, radioactive-iodine-non-responsive, recurrent or metastatic, well-differentiated thyroid carcinomas (Busaidy and Cabanillas, 2012[7]; Sherman, 2010[68]). 

### Treatment of well-differentiated thyroid carcinomas

Currently, well-differentiated (papillary and follicular) thyroid carcinomas are treated by using a combination of surgery to remove the thyroid and, if necessary, radioactive iodine therapy and THST. It has been reported that patients with stage II or well-differentiated high-risk thyroid carcinoma benefit from radioactive iodine therapy, improving their overall survival (Jonklaas et al., 2006[23]). This is not the case for patients with stage I well-differentiated thyroid carcinomas, whose overall survival worsens when ^131^I is used (Jonklaas et al., 2006[23]). Furthermore, radioactive iodine therapy can also be useful for patients with metastatic disease, in which the distant neoplastic foci have thyroid-like features, responding to treatment and ^131^I uptake (Cabanillas et al., 2016[8]). Thus, although radioactive iodine therapy is efficient in many cases, it has severe side effects associated to it such as off-target organ damage (*i.e.,* salivary glands and bone marrow) and increasing the patients risk of developing hematologic malignancies (Kloos, 2009[29]). 

Furthermore, THST has been shown to be an efficient therapy for the treatment of well-differentiated thyroid carcinomas, increasing the patients' overall survival, reducing disease recurrence and cancer-related mortality (Cabanillas et al., 2016[8]; Jonklaas et al., 2006[23]). Moreover, high-risk well-differentiated thyroid carcinoma patients strongly benefit from this therapy, whose survival can be increased 2 to 3 fold-factor (Sherman, 2003[68]). 

In the case of metastatic and/or advanced well-differentiated thyroid carcinomas, systemic chemotherapies are used. But, unfortunately, these malignancies are associated with poor response rates and short time of response when systemic therapies are used (Pacini et al., 2010[51]). Some of the cytotoxic drugs used for the treatment of advanced thyroid carcinoma include doxorubicin, paclitaxel, tamoxifen, bleomycin, epirubicin, cisplatin and octreotide (Harada et al., 1971[21]; Liebner et al., 2016[36]; Pacini et al., 2010[51]; Sherman, 2003[68], 2010[67]). The choice of systemic therapies is somewhat inconclusive, as reports with different response rates to the same protocols are observed. For example, doxorubicin, the most studied and used chemotherapy agent for these malignancies, seems to be one of the cytotoxic drugs with the highest response rate, ~40 %; but this response is temporary and most responses to this drug are incomplete (Sherman, 2003[68]). Other studies show that some patients only have a response rate of ~17 %, for the single use of doxorubicin, displaying however, an increase in response rates when combined with cisplatin or even achieving complete responses (Lim, 2012[38]; Shimaoka et al., 1985[69]). Altogether, doxorubicin seems to be the most effective in treating metastatic disease, that are non-responsive to other treatments, as well as for medullary thyroid carcinoma, either as a single-agent chemotherapy or in combination with other agents (Liebner et al., 2016[36]). Such combined regimens include: doxorubicin, bleomycin and vincristine; or bleomycin, doxorubicin and cisplatin (Liebner et al., 2016[36]).

When administered at low doses, cytotoxic agents can be used as radiosensitizers for external radiation therapy to increase its efficacy (Lim, 2012[38]). 

### Treatment of anaplastic thyroid carcinoma

According to the most recent American Thyroid Association (ATA) guidelines, to manage and control local and metastatic anaplastic thyroid carcinoma, a combination of surgery, chemotherapy and radiotherapy should be used (Smallridge et al., 2012[72]). Surgery for local disease management should be applied when total or major part of the mass is resectable, without damaging the surrounding vital structures (*i.e.*, trachea, larynx, vocal cords, esophagus, major vessels and nerves, etc.). ATA also describes that only a small percentage of patients fit these criteria. In palliative care, surgery can improve quality of life, for example by decompressing the patient's airway. Regardless of potential-curative or palliative intent of treatment, tracheostomy is recommended by ATA for airway management (Ranganath et al., 2015[55]). As adjuvant or primary course of treatment, radio- and chemotherapy are also used. Different cytotoxic drugs are used, such as regimens combining paclitaxel and carboplatin, or docetaxel and doxorubicin, but multimodal chemotherapy regimens composed of only one cytostatic drug, such as cisplatin, paclitaxel and doxorubicin, are also part of the therapeutic options. In the case of advanced metastatic disease, surgery and radiotherapy are not recommended, being the systemic therapies the most appropriate.

For this, anthracyclines and platins are used as first-line cytotoxic chemotherapy regimens. Overall survival seems to be maximized when the three presented therapeutic options are combined (Molinaro et al., 2017[43]).

Although the above-described therapeutic options are available, both individually and combined, its efficacy in treating and curing patients with anaplastic thyroid carcinoma is very low (Molinaro et al., 2017[43]). For example, although it has been described that total resection of tumor increases 1 year survival to 92 %, the mean survival of patients that undergo surgery is 3.5 months (Smallridge and Copland, 2010[73]). Radiotherapy alone does not seem to improve survival (2.3 months), but in combination with surgery and chemotherapy, survival rates of 2 years after diagnosis, for some patients, are reported (Nagaiah et al., 2011[44]; Smallridge and Copland, 2010[73]). 

Radiotherapy has no effect in disease reoccurrence, with half of the patients relapsing when treated with radiotherapy alone (Smallridge and Copland, 2010[73]). Due to the recurrence rate, cytotoxic agents such as doxorubicin, can also be used as radiosensitizers, improving local long-term control rate (68 %). Although this cytotoxic agent is the most used in chemotherapy regimens for anaplastic thyroid carcinoma, its response rate is around 22 % (Molinaro et al., 2017[43]; Nagaiah et al., 2011[44]; Smallridge and Copland, 2010[73]). Paclitaxel and docetaxel, also used in chemotherapy regimens, show a response rate of around 53 and 14 %, respectively (Molinaro et al., 2017[43]; Nagaiah et al., 2011[44]). Regimens composed of only cisplatin, bleomycin and methotrexate also have poor response rates (Nagaiah et al., 2011[44]). The poor response rates presented might be due to anaplastic thyroid carcinoma expressing multidrug resistance-associated proteins and due to the presence of cancer stem cells (Haghpanah et al., 2016[20]; Nagaiah et al., 2011[44]). Although systemic treatment with chemotherapy has achieved promising results, combining doxorubicin and cisplatin seems to be more effective in reducing mass size, being, for this reason, the standard systemic therapeutic regimen (Molinaro et al., 2017[43]; Smallridge and Copland, 2010[73]).

### Novel therapies

**Figure 1:**
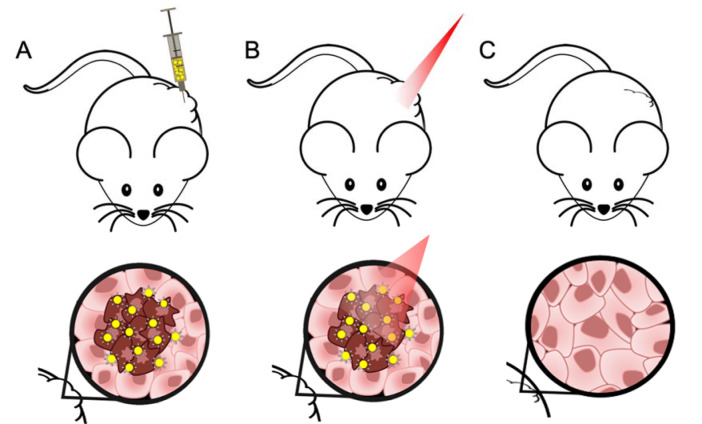
Different steps of PTT using a targeted hybrid gold-based nanosystem developed and a near-infrared (NIR) laser, in a xenograft mice model. (A) In situ administration of hybrid nanosystem at the tumor site, specific for tumor cells (represented in a darker color); (B) Irradiation of the injected site with a NIR laser to activate the formulation; (C) Reduction of tumor mass through thermal ablation.

To overcome the lack of both efficacy and tumor selectivity, nanosystems-based therapeutical approaches have been developed with promising results. One example is the copper-based nanosystems for radiotherapy and Photothermal Therapy (PTT), used to treat thyroid carcinomas (Zhou et al., 2015[86]). PTT is a non-pharmacologic and less invasive approach to target and reduce tumors by thermal ablation of cancer cells, with minimal side effects when compared to other therapeutic options (Silva et al., 2016[71]), as represented in Figure 1(Fig. 1). A gold-based nano hybrid approach has been developed for the treatment of melanoma, also showing very promising results for the treatment of other tumors, such as anaplastic thyroid carcinoma (Silva et al., 2016[70][71]; Amaral, 2020[2]).

Moreover, an incoming therapeutic approach for the treatment of anaplastic thyroid carcinoma is using targeted inhibitors for hyperactive and/or mutant components of signaling pathways (Saini et al., 2018[60]). As previously mentioned, members of the RAF/ MAPK and MEK pathway are mutated in this malignancy (*i.e.,* EGFR, BRAF, Ras) (Saini et al., 2018[60]; Santarpia et al., 2008[62]; Smallridge et al., 2009[74]). Recent studies reported promising results, either of decrease in tumor mass or good responses, using targeted/multi-targeted therapies, both individually or combined, enlightening the benefits of using sequencing tools to identify possible targets for each patient (Tiedje et al., 2018[81]). Furthermore, small-molecule tyrosine kinase inhibitors, such as sorafenib (Nagaiah et al., 2011[44]), vemurafenib (Rosove et al., 2013[57]), dabrafenib (Subbiah et al., 2018[79]), and trametinib (Subbiah et al., 2018[79]), have been shown to be promising for the treatment of anaplastic thyroid carcinoma, being currently in clinical trials, alone or in combination (*i.e*., dabrafenib + trametinib) (Nagaiah et al., 2011[44]; Subbiah et al., 2018[79]; Tiedje et al., 2018[81]). However, when a small-molecule tyrosine kinase inhibitor was used alone, disease reoccurrence was reported by most patients (Saini et al., 2018[60]). Table 2(Tab. 2) (References in Table 2: Grande et al., 2013[17]; Ha et al., 2010[19]; Lim et al., 2013[37]; Rosove et al., 2013[57]; Subbiah et al., 2018[79]; Tahara et al., 2017[80]) summarizes the promising results of completed clinical trials using small-molecule tyrosine kinase inhibitors for the treatment of anaplastic thyroid carcinoma.

Some of these therapeutic results displayed much higher response rates in comparison to the current therapies used, being 10 months the longest overall survival rate reported. The 2-3-fold increase in overall survival corresponds to less than a year.

Table 3(Tab. 3) resents ongoing clinical trials (Phase I and II) registered on the ClinicalTrials.gov database, and its respective database ID, being conducted worldwide, using different treatment approaches and protocols (*i.e.,* radiotherapy, chemotherapy, immunotherapy, small-molecule tyrosine kinase inhibitors, and a combination of these therapies) for the treatment of anaplastic thyroid carcinoma.

## Conclusion

Although some advances have been done regarding the treatment of anaplastic thyroid carcinoma, it still presents low survival rates. There is an urgent need to improve the treatment of this rare malignancy, in order to significantly increase patients' survival and their life quality improvement. Altogether, the best approach seems to be a more personalized multimodal course of treatment, since there is considerable variability of response to treatments using targeted/multi-targeted therapies between individuals with tumor molecular profile. 

## Funding

This research was funded by Fundação para a Ciência e a Tecnologia (FCT) through the Project Reference UID/DTP/04138/2019. 

## Conflict of interest

The authors declare that they have no conflict of interest.

## Figures and Tables

**Table 1:**
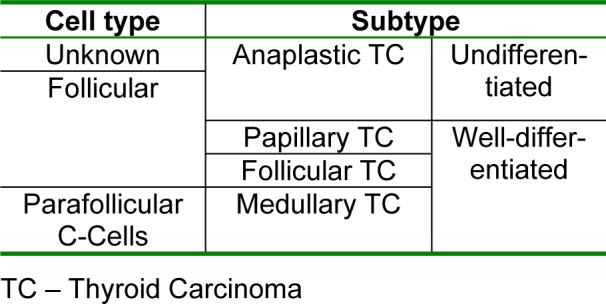
Classification of thyroid carcinomas

**Table 2:**
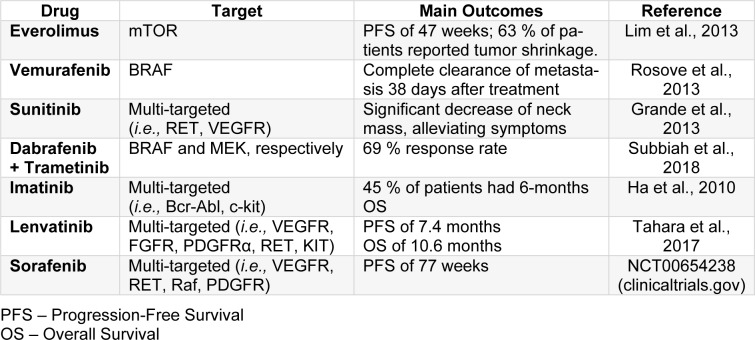
Results of completed clinical trials using targeted or multi-target therapies for the treatment of anaplastic thyroid carcinoma

**Table 3:**
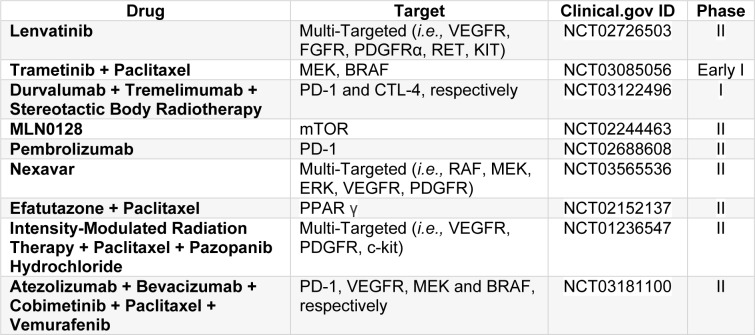
Ongoing clinical trials using targeted or multi-targeted therapies, radiotherapy, immunotherapy and chemotherapy for the treatment of anaplastic thyroid carcinoma
